# Temperature control in adults after cardiac arrest: a survey of current clinical practice in Germany

**DOI:** 10.1186/s13054-023-04319-7

**Published:** 2023-01-23

**Authors:** Kevin Roedl, Sebastian Wolfrum, Guido Michels, Martin Pin, Gerold Söffker, Uwe Janssens, Stefan Kluge

**Affiliations:** 1 Department of Intensive Care Medicine, University Medical Center Hamburg-Eppendorf, Martinistraße 52, 20246 Hamburg, Germany; 2 Emergency Department, University of Luebeck, Luebeck, Germany; 3 Department of Acute and Emergency Care, St.-Antonius-Hospital, Eschweiler, Germany; 4 Emergency Department, Florence-Nightingale Hospital, Duesseldorf, Germany; 5 Medical Clinic and Medical Intensive Care Medicine, St.-Antonius Hospital, Eschweiler, Germany

**Keywords:** Cardiac arrest, Hypothermia, Targeted temperature management, Temperature control, Clinical practice

## Abstract

**Background:**

Temperature control is recommended after out of hospital cardiac arrest (OHCA) by international guidelines. This survey aimed to investigate current clinical practice and areas of uncertainty.

**Methods:**

Online survey targeting members of three medical emergency and critical care societies in Germany (April 21–June 6, 2022) assessing post-cardiac arrest temperature control management.

**Results:**

Of 341 completed questionnaires 28% (*n* = 97) used temperature control with normothermic target and 72% (*n* = 244) temperature control with hypothermic target. The definition of fever regarding patients with cardiac arrest ranged from ≥ 37.7 to 39.0 °C. Temperature control was mainly started in the ICU (80%, *n* = 273) and most commonly core cooling (74%, *n* = 254) and surface cooling (39%, *n* = 134) with feedback were used. Temperature control was maintained for 24 h in 18% (*n* = 61), 48 h in 28% (*n* = 94), 72 h in 42% (*n* = 143) and longer than 72 h in 13% (*n* = 43). 7% (n = 24) were using different protocols for OHCA with initial shockable and non-shockable rhythm. Additional 14% (*n* = 48) were using different temperature control protocols after in-hospital cardiac arrest (IHCA) compared with OHCA. Overall, 37% (*n* = 127) changed practice after the publication of the ERC-2021 guidelines and 33% (*n* = 114) after the recent publication of the revised ERC-ESICM guideline on temperature control.

**Conclusions:**

One-third of the respondents changed clinical practice since recent guideline update. However, a majority of physicians further trusts in temperature control with a hypothermic target. Of interest, 14% used different temperature control strategies after IHCA compared with OHCA and 7% for shockable and non-shockable initial rhythm. A more individualized approach in post resuscitation care may be warranted.

## Introduction

Mortality rates after cardiac arrest (CA) are high and mainly triggered by post-CA shock and brain injury [1]. As only neuroprotective intervention temperature control with hypothermic target (TCHT) has been recommended in unconscious adults after CA for almost two decades. Since then, studies showed a slow acceptance and implementation process of temperature control (TC) over several years [2]. However, recent randomized trials failed to show improved functional outcome when compared with strict normothermia [3, 4].

Unconscious adults after CA should be treated by TC to actively prevent fever in accordance with the most recent European Resuscitation Council (ERC) and European Society of Intensive Care Medicine (ESICM) guidelines [5]. However, optimal target temperature, methods for TC as well as optimal duration of TC and if there might be sub-groups benefiting from TCHT are unknown and still under debate.

To date, current surveys regarding clinical practice of TC after in- and out-of-hospital CA following the publication of the latest ERC-ESICM guidelines are missing. Therefore, an online based cross-sectional survey among members of three emergency and critical care societies was performed.

## Methods

### Study design and survey development

Cross-sectional anonymous electronic survey focusing on current clinical practice of TC across Germany. The standardized questionnaire was developed and reviewed by leading members of three German emergency and critical care societies using Uni-Park software (https://www.unipark.com/). The questions were built based on recent literature/guidelines [5]. Multiple-choice and free-text questions were used to allow comprehensive detailed information. The survey had 43 questions, 5 sections, and required 10 min on average to be completed. The survey was pre-tested with 6 specialized physicians to check for clarity and validity. Overall, Part A included demographic of participants and general CA characteristics; Part B reflected TC practice; Part C reflected practice after IHCA and OHCA; Part D included TC in E-CPR; Part E focused on changes in practice after recent guidelines.

### Survey participants and data collection

The survey targeted emergency physicians and intensivists directly involved in the post-CA care. Three German societies (German Society for Medical Intensive Care Medicine (DGIIN), German Society of Cardiology (DGK), German Society for Interdisciplinary Emergency and Acute Care Medicine (DGINA)) and their specific sections dealing with CA management distributed the survey among their members via email. To maximize the response, three reminders were sent. No identifiable data were collected, and consent was implied by completing the survey. The survey was online from April 21 to June 6, 2022. This survey was approved by the local ethics committee (2022-300183-WF).

### Statistical analysis

Raw data were checked for data completeness and potential duplicates. Statistical analysis was conducted using IBM SPSS Statistics Version 24.0 (IBM Corp., Armonk, NY) and graphical presentation was conducted using GraphPad Prism (Version 9.1.0, GraphPad Software, San Diego, California, USA). Descriptive statistics was used to present the data. Data are presented as count and relative frequency or median and 25–75% interquartile range (IQR). Variables were compared by Chi-squared, Fisher exact or Mann–Whitney *U* test as appropriate. Generally, a *p*-value < 0.05 was considered statistically significant.

## Results

Overall, 341 respondents completed the survey. Those were primary working as intensivists (54%, *n* = 183), cardiologists (18%, *n* = 61) or emergency physicians (16%, *n* = 54).

### Temperature control practice after cardiac arrest

**Fig. 1:**
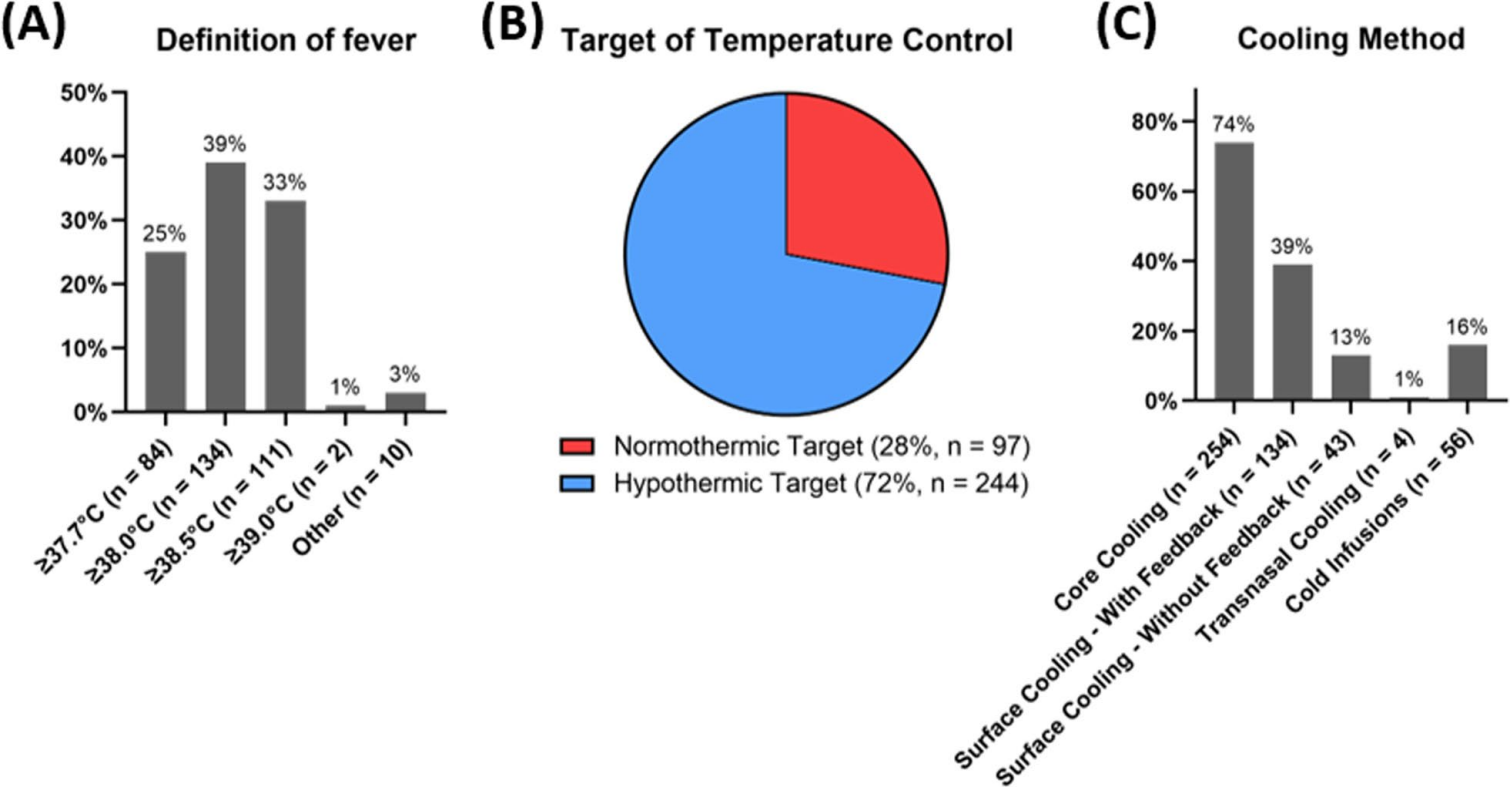
**A** Definition of fever used in patients with cardiac arrest; **B** Temperature control target-stratified according temperature control with normothermic and hypothermic target; **C** Cooling methods used by the survey participants

Different definitions of fever are shown in Fig. 1A. 28% (*n* = 97) reported to use temperature control with a normothermic target (TCNT) and 72% (*n* = 244) TCHT (Fig. 1B). Those using TCHT, 64% (*n* = 156) targeted 32–34 °C and 36% (*n* = 88) 34–36 °C. Initiation of TC is mainly started within the ICU in 80% (*n* = 273). Table 1 reports the characteristics of clinical practice regarding different targeted temperature levels.

**Table 1:** Characteristics of clinical practice stratified according temperature control: temperature control with normothermic target and temperature control with hypothermic target (32–34 °C vs. 34–36 °C)

Variables	TC normothermic target (*n* = 97)	TC (32–34 °C) (*n* = 156)	TC (34–36 °C) (*n* = 88)	*p*-value (Normothermia vs. Hypothermia)
*OHCA treated per year*				
≤ 50	30 (31)	43 (28)	28 (32)	0.12
51–100	38 (39)	73 (47)	49 (56)	
> 100	29 (30)	40 (26)	11 (13)	
*Size of hospital*				
≤ 100 beds	0 (0)	1 (1)	0 (0)	0.88
100–200 beds	8 (8)	13 (8)	6 (7)	
200–500 beds	29 (30)	55 (35)	27 (31)	
500–800 beds	20 (21)	31 (20)	22 (25)	
≥ 800 beds	40 (41)	56 (36)	33 (38)	
*Cardiac arrest center*				
Certified cardiac arrets center	46 (47)	62 (40)	31 (35)	0.23
Certification planned (< 1 year)	14 (14)	28 (18)	21 (24)	
Not certified and not planned	37 (38)	66 (42)	36 (41)	
*Duration hypothermic target temperature*				
24 h	–	136 (87)	72 (82)	–
48 h	–	16 (10)	7 (8)	
72 h	–	4 (3)	9 (10)	
*Duration of temperature control*				
24 h	14 (14)	30 (19)	17 (19)	< 0.001
48 h	23 (24)	42 (27)	29 (33)	
72 h	58 (60)	54 (35)	31 (35)	
> 72 h	2 (2)	30 (19)	11 (13)	
*Rewarming rate*				
0.25 °C/h	–	94 (60)	43 (49)	–
0.5 °C/h	–	35 (22)	19 (22)	
No controlled rewarming	–	6 (4)	16 (18)	
Other	–	21 (13)	10 (11)	
*Cooling methods*				
Core cooling	68 (70)	120 (77)	66 (75)	0.24
Surface cooling-with feedback	43 (44)	59 (38)	32 (36)	0.23
Surface cooling-without feedback	24 (25)	11 (7)	8 (9)	< 0.001
Transnasal cooling	2 (2)	2 (1)	0 (0)	0.34
Cold infusions	25 (26)	21 (13)	10 (11)	< 0.05

Data are expressed as *n* (%) or median (interquartile range, IQR 25/75%)

OHCA, out-of-hospital cardiac arrest; TC, temperature control

### Differences in temperature control practice regarding initial rhythm and IHCA

93% (*n* = 317) were using the same TC protocol after initial shockable and non-shockable rhythm after OHCA. Those with different protocols were using no TC (*n* = 7), an individual strategy (*n* = 7), a different length/duration of TC (*n* = 5) or other (*n* = 5). After IHCA 86% (*n* = 293) of participants were using the same protocol as for OHCA. A different protocol included no TC (*n* = 8), an individual approach (*n* = 27), different length of TC (*n* = 1) and other unspecified changes (*n* = 12) compared to OHCA.

### Practice in extracorporeal cardiopulmonary resuscitation

Forty-five per-cent (*n* = 155) are using E-CPR as resuscitation strategy. All were using TC after E-CPR. Targeted temperature was TCHT (32–34 °C: 43 (*n* = 66), 34–36 °C: 41% (*n* = 63)) and TCNT (17%, *n* = 26). The duration of TCHT was 24 h in 68% (*n* = 88), 48 h in 12% (*n* = 16), 72 h in 17% (*n* = 22), 96 h in 1% (*n* = 1) and other in 1% (*n* = 2). The total time of TC was 24 h in 6% (*n* = 10), 48 h in 12% (*n* = 19), 72 h in 65% (*n* = 100), 96 h in 10% (*n* = 16), > 96 h in 4% (*n* = 6) and other in 3% (*n* = 4), respectively. Methods for TC were ECLS (76%, *n* = 118), core cooling (39%, *n* = 60), external cooling (22%, *n* = 34) and other (2%, *n* = 3). Twenty-five per-cent (*n* = 39) reported different TC strategies in E-CPR.

### Change in practice

37% (*n* = 127) changed clinical practice after publication of ERC-2021 guidelines. Furthermore, 33% (*n* = 114) changed practice after publication of the revised ERC-ESICM guidelines on TC after CA.

## Discussion

This online survey among emergency and medical critical care physicians found that the majority of physicians is using TCHT and further trust in the effectiveness of hypothermia. Only one-third changed clinical practice following the recent guideline update. Of interest, 14% reported to use a different TC strategy after IHCA compared with OHCA.

Current international guidelines recommend actively prevention of fever (≥ 37.7 °C) after CA in adults [5, 6]. Occurrence of fever in CA survivors was shown to be associated with unfavourable neurological outcome [7]. However, the definition of normothermia in humans remains uncertain. We found that only one-quarter of respondents defined fever in accordance with recent guidelines. Alternative definitions could be associated with the fact of high cost of cooling devices. When normothermia is targeted after CA the use of protocols aiming to avoid poor TC must be mandatory. Poor implementation of TC may lead to further patient harm and deleterious effects on functional outcome in CA survivors.

Therapeutic hypothermia was first recommended by ILCOR 2003[8]. Since its first introduction in guidelines a slow process of implementation into clinical practice followed [2, 9, 10]. Recent studies did not show differences regarding strict normothermia and TCHT [3, 4]. Current recommendations do not speak for or against the use TCHT (32–36 °C), it is up to bedside clinicians which strategy to use in CA survivors [5]. A change in practice after ERC-2021 and the recent ERC-ESICM guideline update was observed in 37% and 33%, respectively. To date, the optimal strategy of TC is unknown. It is unclear if only using pharmacological measures are effective enough or specific cooling devices must be applied upfront. The survey revealed that most of the respondents were using core or surface cooling devices with feedback. The optimal duration for TCHT is unknown although the period of hypothermia is most commonly 24 h and fever prevention is recommended for at least 72 h [5, 6]. Of interest, 15% of the cohort were using a longer duration of TCHT. Although otherwise recommended 9% were using no controlled rewarming which could lead to rebound hyperthermia which is associated with worse outcome [11].

TC is recommended regardless of the initial rhythm and location. However, we observed two interesting findings. Firstly, 7% of respondents reported to apply a different protocol to patients with initial shockable rhythm compared with initial non-shockable rhythm. Some were using no TC after non-shockable rhythm, the majority a different length or an individual temperature control approach. This is of importance, because the HYPERION-trial found a benefit of TCHT in patients with initial non-shockable rhythm [12]. Secondly, 14% report to use a different protocol after IHCA compared with OHCA. Of interest, the majority (56%) was using an individual (patient personalized) TC approach and 17% no TC. Among others the largest observational study of the GTWG-Resuscitation registry questioned the effectiveness of TC in IHCA [13, 14]. The HYPERION-trial found improved functional outcome after IHCA [12, 15]. One recent randomized trial did not show differences regarding mortality or functional outcome using TCHT compared to normothermia after IHCA [4]. However, there is limited evidence concerning the potential benefit of TCHT after non-shockable rhythm and IHCA. E-CPR as alternative resuscitation strategy in refractory CA is more and more commonly used [16, 17]. 83% used TCHT in E-CPR and the duration of TCHT was > 24 h in 32%. Overall, 25% of respondents apply a different protocol to E-CPR patients. This might be explained by a higher chance of brain damage in this group and potential positive effects of TCHT. In general, a more personalized approach for patients including location of arrest, initial rhythm and E-CPR maybe warranted.

This study has limitations. First, we show results of an online survey of primarily targeting emergency and medical critical care physicians and may only reflect practice in Germany. Second, the study included a medium sample size and was designed as online survey with its naturally rigid structure. Third, the possibility of responder bias could not be avoided, being inherent to questionnaires of this kind.

## Conclusions

This study provides important insights regarding current post-CA TC practice. There is a large variation in TC practice among respondents reflecting uncertainties and knowledge gaps in post-CA care. One-third of the respondents changed clinical practice since recent guideline update. However, a majority of physicians further trusts in TCHT. Of interest, 14% used different temperature control strategies after IHCA compared with OHCA.

## Data Availability

The datasets supporting the conclusions of this article are included within the article.
